# Human mesenchymal stem cells possess different biological characteristics but do not change their therapeutic potential when cultured in serum free medium

**DOI:** 10.1186/scrt522

**Published:** 2014-12-04

**Authors:** Youwei Wang, Hehe Wu, Zhouxin Yang, Ying Chi, Lei Meng, Aibin Mao, Shulin Yan, Shanshan Hu, Jianzhong Zhang, Yun Zhang, Wenbo Yu, Yue Ma, Tao Li, Yan Cheng, Yongjuan Wang, Shanshan Wang, Jing Liu, Jingwen Han, Caiyun Li, Li Liu, Jian Xu, Zhi Bo Han, Zhong Chao Han

**Affiliations:** State Key Laboratory of Experimental Hematology, National Engineering Research Center of Stem Cells, Institute of Hematology and Hospital of Blood Diseases, Chinese Academy of Medical Science & Peking Union Medical College, 288 Nanjing Road, 300020 Tianjin, China; National Engineering Research Center of Cell Products/AmCellGene Co. Ltd, 80 Fourth Street in Tianjin Economic and Technological Development Zone Block B3, 300457 Tianjin, China; TEDA Life Science and Technology Research Center, Institute of Hematology, Chinese Academy of Medical Science, 80 Fourth Street in Tianjin Economic and Technological Development Zone Block B4, 300457 Tianjin, China

## Abstract

**Introduction:**

Mesenchymal stem cells (MSCs) are widely investigated in clinical researches to treat various diseases. Classic culture medium for MSCs, even for clinical use, contains fetal bovine serum. The serum-containing medium (SCM) seems a major obstacle for MSCs-related therapies due to the risk of contamination of infectious pathogens. Some studies showed that MSCs could be expanded in serum free medium (SFM); however, whether SFM would change the biological characteristics and safety issues of MSCs has not been well answered.

**Methods:**

Human umbilical cord mesenchymal stem cells (hUC-MSCs) were cultured in a chemical defined serum free medium. Growth, multipotency, surface antigen expression, telomerase, immunosuppressive ability, gene expression profile and genomic stability of hUC-MSCs cultured in SFM and SCM were analyzed and compared side by side.

**Results:**

hUC-MSCs propagated more slowly and senesce ultimately in SFM. SFM-expanded hUC-MSCs were different from SCM-expanded hUC-MSCs in growth rate, telomerase, gene expression profile. However, SFM-expanded hUC-MSCs maintained multipotency and the profile of surface antigen which were used to define human MSCs. Both SFM- and SCM-expanded hUC-MSCs gained copy number variation (CNV) in long-term *in vitro* culture.

**Conclusion:**

hUC-MCSs could be expanded in SFM safely to obtain enough cells for clinical application, meeting the basic criteria for human mesenchymal stem cells. hUC-MSCs cultured in SFM were distinct from hUC-MSCs cultured in SCM, yet they remained therapeutic potentials for future regenerative medicine.

**Electronic supplementary material:**

The online version of this article (doi:10.1186/scrt522) contains supplementary material, which is available to authorized users.

## Introduction

Mesenchymal stem cells (MSCs) possess the ability of self-renew and multipotency. These cells can replicate *in vitro* and have the potential to differentiate to bone, fat and cartilage tissues [1]. Numerous preclinical and clinical studies have demonstrated that MSCs hold an alluring prospect as cellular therapies, based on their multipotency, hematopoietic-supporting and immunosuppressive abilities. MSC-based tissue-engineering approaches could treat patients with long bone defects [2, 3]. Co-transplantation of MSCs with HLA-disparate hematopoietic stem cells could accelerate lymphocyte recovery and reduce the risk of graft failure [4]. For the small effect on T-cell responses to pathogens, infusion of MSCs suppressed alloantigen-induced T-cell function and might be a promising therapy for graft-versus-host disease [5, 6]. MSCs, described as a very rare population in bone marrow by Friedenstein and colleagues [7], need to be expanded *in vitro* to achieve the amount required for administration. Several safety-related issues have been of wide concern in clinical applications of *in vitro* expanded MSCs. It is not clear whether MSCs could maintain genomic stability during expansion *in vitro* and whether injection of MSCs could lead to cancer *in vivo*.

Many studies have demonstrated that MSCs will not undergo malignant transformation after long-term *in vitro* culture in serum-containing culture [8]. Other studies on pluripotent stem cells have revealed that the number of chromosomes and the copy number of specific regions in the genome of embryonic stem cells or induced pluripotent stem cells could mutate in the process of *in vitro* expansion [9–14]. Similarly, copy number variation (CNV) was found in adipose tissue-derived MSCs [15] after long-term culture, even though they did not undergo malignant transformation. Previous studies paid much attention to the safety issues of MSCs cultured in serum-containing medium (SCM) [16]. However, it is not desirable to prepare MSCs for clinical application in SCM. The utmost problem associated with bovine and human serum is the safety issue. Bovine serum might contain zoonotic viruses (including prion), which cannot be cleaned up during the process of preparing MSCs for clinical use. Human serum might contain undetectable pathogen, which could easily spread between human beings during stem cell transplantation. From this perspective, human serum is more dangerous than serum of animals. In recent years, some human serum or human platelet lysate products are solvent/detergent treated, which makes them much less likely to transmit an infectious disease, without deleting the risk completely [17].

In addition, serum is ill-defined, has a high degree of batch-to-batch variation, is hard to standardize and can harm the process control and stability of quality and production. Serum-free medium (SFM) is an ideal system for cellular therapy. MSCs expanded in SFM perform much better in quality control and stability. Many previous studies focused on increasing attachment and growth of MSCs in SFM [18]. Other studies evaluated the clinical application related biological characteristics of SFM-expanded MSCs [19, 20]. However, the safety and efficacy of MSCs cultured in SFM have not been well evaluated [21]. In this study we investigated whether human umbilical cord mesenchymal stem cells (hUC-MSCs) expanded in SFM change their biological characteristics and clinical safety-related issues, which included genome and transcriptome stability.

## Methods

### Growth characteristics of MSCs in serum-free medium

hUC-MSCs derived from five different donors were isolated from Wharton’s jelly by enzymatic digestion [22] and frozen in a master cell bank after short-term expansion in SCM. This study is approved by the Institutional Review Board of the Chinese Academy of Medical Science and Peking Union Medical College. Umbilical cords were obtained following the ethical guidelines with written informed consent from donors. All experimental research of this study was in compliance with the Helsinki Declaration. After recovery from the master cell bank, hUC-MSCs were cultured on a tissue culture surface with SCM that contained 10% fetal bovine serum (ExCell Bio, Shanghai, China) or on a chemically treated cell culture surface (CellBIND; Corning Incorporated, Corning, NY, USA) with a chemically defined SFM (MSCGM-CD; Lonza, Walkersville, MD, USA), at 37°C and 5% carbon dioxide. After reaching 90% confluence, hUC-MSCs were detached and subcultured at a ratio of 1:3 until reaching senescence. The time needed to obtain confluence for every passage was recorded to calculate the population-doubling time. β-galactosidase were analyzed at late passage by a cellular senescence assay kit (Millipore, Billerica, MA, USA) following the manufacturer’s protocol. In this assay, senescent cells were stained as a distinctive blue color.

### Differentiation of MSCs cultured in serum-free medium

For osteogenic and adipogenic differentiation, SFM-expanded hUC-MSCs at the 10th passage were seeded in 24-well plates at a concentration of 5 × 10^4^ cells per well. The StemPro Adipogenesis Differentiation Kit (A10070-01; GIBCO, Grand Island, NY, USA) and the Osteogenesis Differentiation Kit (A10072-01; GIBCO) were used as the differentiation-inducing medium. The medium was refreshed twice every week. After 21 days of differentiation, cells were fixed in 70% ethanol and stained with Alizarin Red S (for osteogenic differentiation) or Oil Red O (for adipogenic differentiation). For chondrogenic differentiation, 4 × 10^5^ SFM-expanded hUC-MSCs at the 10th passage were suspended in 1 ml StemPro Chondrogenesis Differentiation Kit (A10071-01; GIBCO) and distributed to 15 ml centrifuge tubes. Cells were centrifuged at 500 × *g* for 5 minutes and then placed in an incubator with the caps loosened. The chondrogenic culture was refreshed twice every week. After 21 days of chondrogenesis, the pellets were fixed in 4% formaldehyde, cut to 5 μm and stained by Toluidine blue.

### Flow cytometric analysis

hUC-MSCs expanded in SFM at the 10th passage were characterized by flow cytometric analysis for specific antigens. For the analysis of cell surface markers, hUC-MSCs expanded in SFM were harvested after detachment, washed in phosphate-buffered saline and then incubated with phcoerythrin-labeled or fluorescein isothiocyanate-labeled monoclonal antibodies against CD14 (12-0149-42; eBioscience, San Diego, CA, USA), CD19 (555413; BD Biosciences, Franklin Lakes, NJ, USA), CD34 (555821; BD Biosciences), CD45 (103105; BioLegend, San Diego, CA, USA), CD73 (550257; BD Biosciences), CD90 (555596; BD Biosciences), CD105 (560839; BD Biosciences), HLA-ABC (555552; BD Biosciences) and HLA-DR (555812; BD Biosciences). For analyzing the expression of Nestin, which is an intracellular marker, SFM-expanded hUC-MSCs were fixed and permeabilized by the Cytofix/Cytoperm™ Fixation/Permeabilization Kit (554714; BD Biosciences). The cells were then stained by phcoerythrin-conjugated antibodies against Nestin. CellQuest was used to perform the analysis on FACS Calibur’ (BD Biosciences, San Jose, CA, USA).

### Telomerase reverse transcriptase analysis

RNA was extracted from SCM-expanded and SFM-expanded hUC-MSCs in SFM using the E.Z.N.A Total RNA Kit (Omega Bio-Tek, Norcross, GA, USA). cDNA was synthesized by M-MLV Reverse Transcriptase (Invitrogen, Carlsbad, CA, USA). TaqMan-based real-time quantitative PCR assay was used to analyze the expression of *hTERT* (the human telomerase catalytic subunit gene) [23]. For each PCR run, a 20 μl reaction mix was prepared with 10 μl TaqMan Gene Expression Master Mix (2×; Applied Biosystems, Warrington, UK), 1 μl of 10 μM upper primer, 1 μl of 10 μM lower primer, 1 μl of 10 μM probe, 1 μl of cDNA and 6 μl ddH_2_0. The reaction mixes were then placed in Applied Biosystems real-time PCR System 7300 with the following thermal cycling conditions: 50°C for 2 minutes, 95°C for 10 minutes, 55 cycles including 95°C for 15 seconds and 60°C for 1 minute. *RPLP0* was used as a reference gene. cDNA prepared from HeLa cells was used as a positive control.

### Immunoregulation analysis

We used lymphocyte proliferation and interferon gamma (IFNγ) analysis, which was described by previous studies [24, 25], to evaluate the immunosuppressive ability of hUC-MSCs after culture in SFM. In brief, SCM-derived and SFM-derived hUC-MSCs were irradiated (60 Gy) and then cultured in 96-well cell culture plates. Two hours later, human peripheral blood mononuclear cells (hPBMCs) were added to hUC-MSCs and cultured with PHA (Sigma, St. Louis, MO, USA) and interleukin-2 (Peprotech, Rocky Hill, NJ, USA). hPBMCs were co-cultured with hUC-MSCs for 3 days. BrdU was added 18 hours before detection. Cell proliferation was measured by BrdU incorporation assay (Roche, Mannheim, Germany). Supernatant were harvested for IFNγ analysis (eBioscience).

### Genetic stability of MSCs cultured in SFM and in SCM

Two pairs of samples that expanded in SFM and SCM were sent to CapitalBio Co. (Beijing, China) for array-based comparative genomic hybridization (aCGH) analysis following the protocol described on the website of CapitalBio Co. [26]. Briefly, the genomic DNA was extracted and purified from hUC-MSCs by phenol–chloroform. According to the quality control requirements of NimbleGen (Madison, WI, USA), the genomic DNA was undegraded and has A260/A280 ≥ 1.8 and A260/A230 ≥ 1.9. NimbleGen human whole-genome tiling arrays containing up to 4.2 million probes, spanning the human genome with a median probe spacing of 2,509 base pairs (Human CGH 3x720K Whole-Genome Tiling v3.0 Array, 05547717001; NimbleGen), were utilized for aCGH analysis. Genomic DNA extracted from SCM-expanded or SFM-expanded hUC-MSCs at the 10th passage was labeled with Cy3. DNA isolated from hUC-MSCs at the third passage, derived from the same donors as those from whom serum-free culture or serum-containing culture were established, were labeled with Cy5 and used as reference samples.

Test (10th passage) and reference (3rd passage) samples were co-hybridized onto arrays and scanned using a MS200 scanner (NimbleGen) with 2 μm resolution. Cy3 and Cy5 signal intensities were computed and normalized using the q-spline method [27]. Segments with |mean log_2_ ratio| ≥ 0.25 and at least five consecutive probes were scored as aberrant DNA copy number changes.

### Transcriptome analysis

Three pairs of hUC-MSC samples, before and after expansion in SFM, were sent to CapitalBio Co. for mRNA microarray analysis. Total RNA was extracted and then qualified by formaldehyde agarose gel electrophoresis. After being quantitated spectrophotometrically, 1 μg total RNA was used to synthesize double-stranded cDNA and then produced biotin-tagged cRNA using the MessageAmp™ II aRNA Amplification Kit (Invitrogen). According to the protocol from Affymetrix (Santa Clara, CA, USA), bio-tagged cRNA was fragmented and hybridized to Human Genome U133 Plus 2.0 containing over 47,000 transcripts. After hybridizing, the arrays were washed, stained by Affymetrix Fluidics Station 450 and scanned by GeneChip Scanner 3000. The intensity values of different microarray were normalized and log_2_ transformed using the RMA gene core algorithm provided by the Expression Console (Affymetrix). Genes with at least twofold changes were selected for further analysis. The Molecule Annotation System (online analysis system provided by CapitalBio Co.) was used to perform Gene Ontology and pathway analysis. Microarray data were deposited into a public database [Gene Expression Omnibus:GSE62665].

### Real-time PCR analysis

Real-time PCR was used to validate the data obtained from the mRNA microarray. Twelve differentially expressed genes including six upregulated genes and six downregulated genes were selected for validation by real-time PCR. These 12 genes included genes related to the cell cycle pathway, histone, cytokine, *MAPK8* and *PIK3R1*. *MAPK8* and *PIK3R1* were selected because they implicated in a large number of pathways. GoTaq Green Master Mix (Promega, Madison, WI, USA) was used for real-time PCR in the 7300 real-time PCR System (Applied Biosystems). Relative expression levels were calculated using ΔΔC_T_ method. Seven housekeeping genes were selected as the candidates of reference genes, and we used geNorm [28] for choosing the most stably expressed housekeeping genes as reference genes for data analysis.

## Results

### Growth characteristics of MSCs in serum-free medium

**Figure 1:**
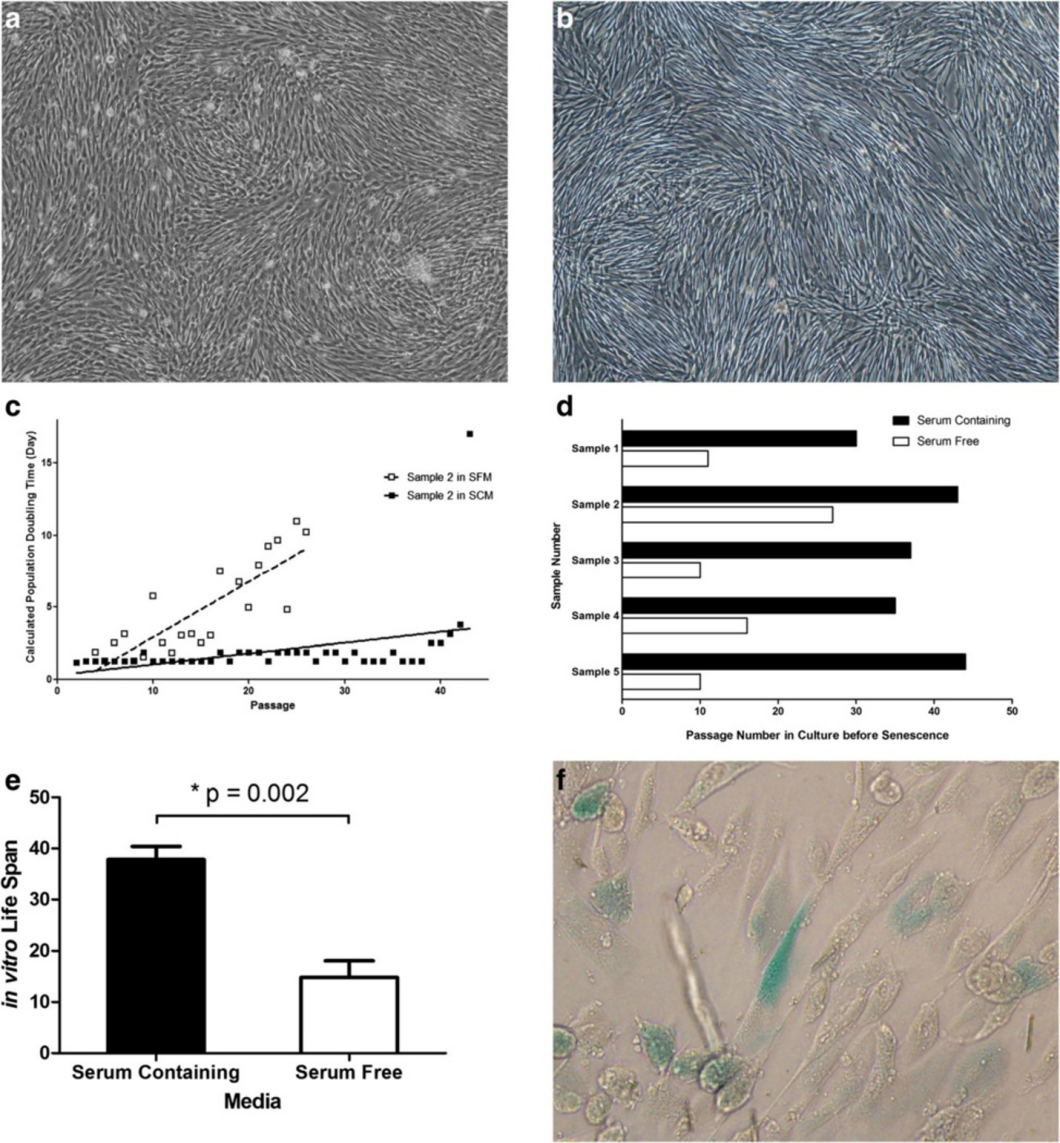
***In vitro*** **growth characteristics of human umbilical cord mesenchymal stem cells cultured in serum-free medium.** Both in SCM **(a)** and in SFM **(b)**, human umbilical cord mesenchymal stem cells (hUC-MSCs) maintained fibroblast-like morphology (40×). **(c)** Calculated population-doubling time. Open boxes, hUC-MSCs expanded in SFM; filled boxes, hUC-MSCs expanded in serum-containing medium (SCM). SFM-expanded hUC-MSCs possessed a much longer calculated population-doubling time. **(d)** *In vitro* lifespan of hUC-MSCs derived from five different donors. **(e)** Paired *t* test was used to compare the lifespan of hUC-MSCs cultured in SFM and SCM. A significant different lifespan between SFM-derived and SCM-derived hUC-MSCs was observed. **(f)** Senescence-associated β-galactosidase activity analysis of SFM-expanded hUC-MSCs at late passage. Blue stain shows senescent cells (×200).

As shown in Figure 1a,b, hUC-MSCs maintained a fibroblastoid appearance in SFM, without noticeable morphological difference to cells expanded in SCM. The major difference between SFM-cultured and SCM-cultured hUC-MSCs was the growth rate and proliferating lifespan *in vitro*. Compared with hUC-MSCs expanded in SCM, the population-doubling time of hUC-MSCs cultured in SFM was prolonged significantly, which meant hUC-MSCs proliferated more slowly in SFM. The population-doubling time of SCM-cultured hUC-MSCs was maintained relatively constant, which was shorter than 2 days before the 20th passage (Figure 1c). The population-doubling time of SFM-cultured hUC-MSCs was very unsteady, which increased with passaging until the senescent stage.

Four out of five hUC-MSCs in this study showed a much shorter lifespan in SFM compared with that in SCM (Figure 1d). They entered the senescence phase between the 10th and 16th passages, which was equivalent to approximately 15 to 20 population doublings. Only one sample displayed a better proliferating capacity in SFM, which reached the senescence phase at the 26th passage. In SCM, all hUC-MSCs in this study did not show any senescent sign before the 30th passage (approximately 47 population doublings).

Actually, we tested at least three different commercial SFMs. In all of them, hUC-MSCs showed much slower growth rate and shorter *in vitro* lifespan compared with that in SCM (Figure 1e). This implied that SFM for MSCs still needs further modification to meet the requirements of growth support. hUC-MSCs cultured in SFM showed a progressive decrease in growth rate and ultimately achieved senescence (Figure 1f). No immortalization or malignant transformation was observed in the culture of hUC-MSCs in SFM.

### Multipotency of serum-free medium-expanded hUC-MSCs

**Figure 2:**
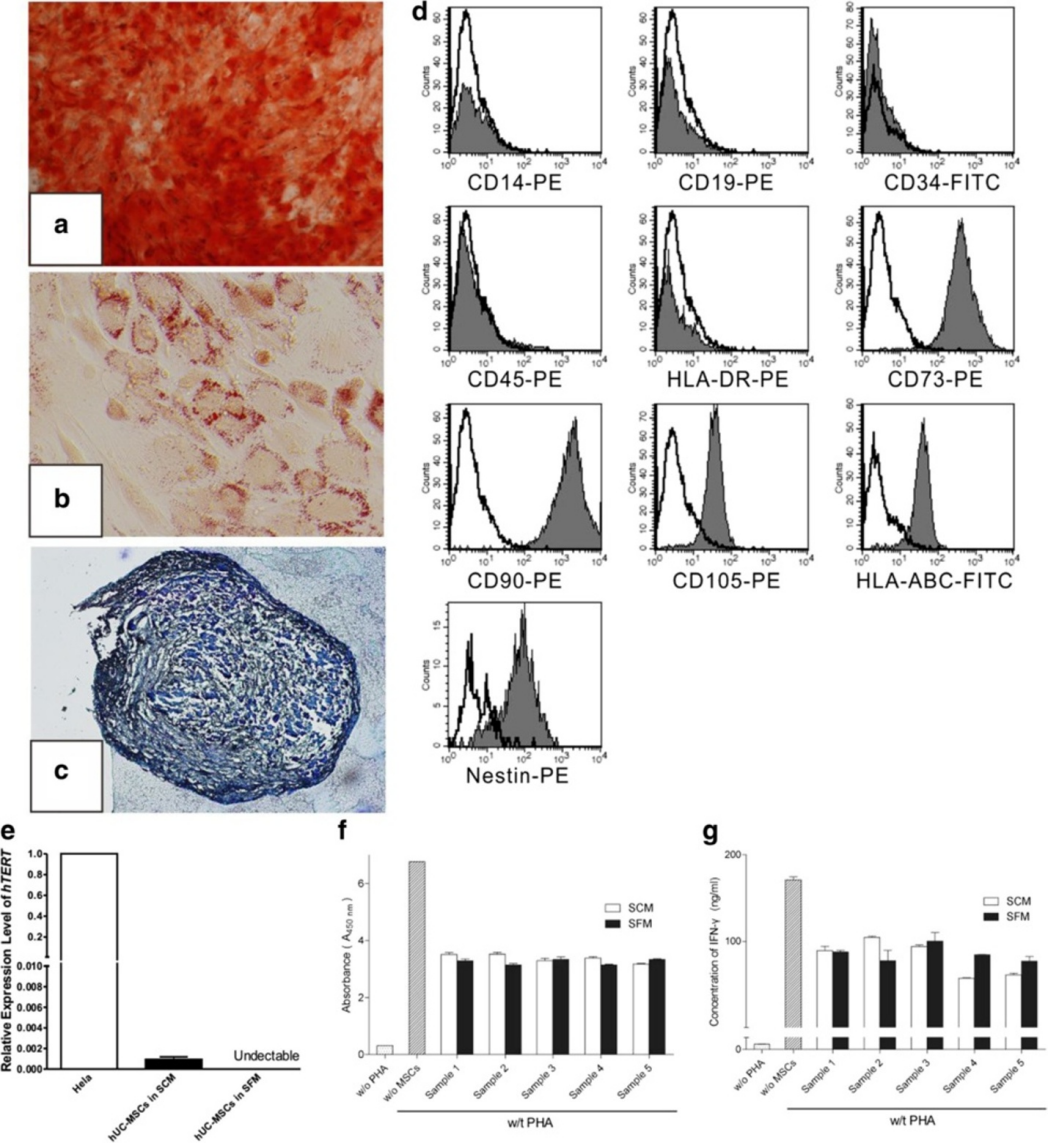
**Induced differentiation, flow cytometric and immunosuppressive ability analysis of human umbilical cord mesenchymal stem cells expanded in serum-free medium.** After differentiation induction, **(a)** osteogenesis was confirmed by Alizarin Red (×40), **(b)** adipogenesis was stained by Oil Red O (×200) and **(c)** chondrogenesis was analyzed by Toluidine Blue (×100). **(d)** Serum-free medium (SFM)-expanded human umbilical cord mesenchymal stem cells (hUC-MSCs) at the 10th passage were labeled with antibodies against human antigens CD14-PE, CD19-PE, CD34-FITC, CD45-PE, CD73-PE, CD90-PE, CD105-PE, HLA-ABC-FITC, HLA-DR-PE and Nestin-PE. **(e)** Expression of *hTERT* in hUC-MSCs. Graph shows the level of *hTERT* transcripts of hUC-MSCs cultured in serum-containing medium (SCM) and SFM (*n* = 5). Values presented as ratio of positive control (HeLa cells). Immunosuppressive ability of hUC-MSCs was evaluated by co-culturing with human peripheral blood mononuclear cells (hPBMCs). **(f)** Proliferation of hPBMCs was quantified based the measurement of BrdU incorporation during DNA synthesis. **(g)** Level of interferon gamma (IFN-γ) in the supernatant was determined by ELISA.

Multipotency of SFM-expanded hUC-MSCs at the 10th passage was evaluated by osteogenic, adipogenic and chondrogenic differentiation. After 21 days of inducing culture, osteogenesis, adipogenesis and chondrogenesis were analyzed by Alizarin Red, Oil Red O and Toluidine Blue separately. As showed in Figure 2a,b,c, SFM-expanded hUC-MSCs differentiate to osteocytes, adipocytes and chondrocytes, which indicates that hUC-MSCs maintained multipotency after *in vitro* expansion in SFM.

### Specific surface antigen expression

Flow cytometric analysis of SFM-expanded hUC-MSCs at the 10th passage is presented in Figure 2d. hUC-MSCs cultured in SFM expressed CD105, CD73, CD90 and HLA-ABC and lacked expression of CD34, CD45, CD14, CD19 and HLA-DR, which met the minimal criteria for identifying human MSCs [29]. In this study, all surface marker expression patterns of SFM-expanded hUC-MSCs were similar to those of SCM-expanded hUC-MSCs. Both SFM-expanded and SCM-expanded hUC-MSCs were positive for Nestin, which were considered to encompass features attributed to hematopoietic niche [30].

### Expression of *hTERT*

Activation or upregulation of *hTERT* is very important for malignant transformation and immortalization of normal human cells [31, 32]. It is therefore necessary to analyze the expression of *hTERT* in MSCs that were cultured in SFM before clinical application. There is controversy about the expression of *hTERT* in MSCs. Based on the analysis of the telomeric-repeat amplification protocol and PCR, Bernardo and colleagues did not find any detectable telomerase in human bone marrow-derived MSCs [33]. However, in Parsch and colleagues’ study, telomerase could be detected in bone marrow MSCs by the telomeric-repeat amplification protocol [34]. Using a TaqMan-based real-time PCR assay, we found that *hTERT* was expressed in hUC-MSCs cultured in SCM at a very low level (Figure 2e). Nevertheless, after culture in SFM, no signal about *hTERT* could be detected even after 55 PCR cycles (Additional file 1). Thus, we claimed that hUC-MSCs possessed expression of *hTERT*, but lost it in SFM.

### Immunosuppressive ability of serum-free medium-expanded hUC-MSCs

To study whether SFM-expanded hUC-MSCs maintained their immunosuppressive ability, we performed an *in vitro* co-culture experiment. First, we analyzed the effect of SFM-expanded hUC-MSCs on inhibiting proliferation of activated hPBMCs by BrdU incorporation assay. Our data showed that SFM-expanded hUC-MSCs could inhibit proliferation of activated hPBMCs (Figure 2f). We then measured the IFNγ concentration in supernatant of the co-culture system by enzyme-linked immunosorbent assay. We found that IFNγ amounts in supernatant were significantly reduced when MSCs were added (Figure 2g). Besides, there was no significant difference between SCM-expanded and SFM-expanded hUC-MSCs on inhibiting proliferation or IFNγ secretion of activated hPBMCs.

### Array-based comparative genomic hybridization analysis of MSCs cultured in SFM and in SCM

**Figure 3:**
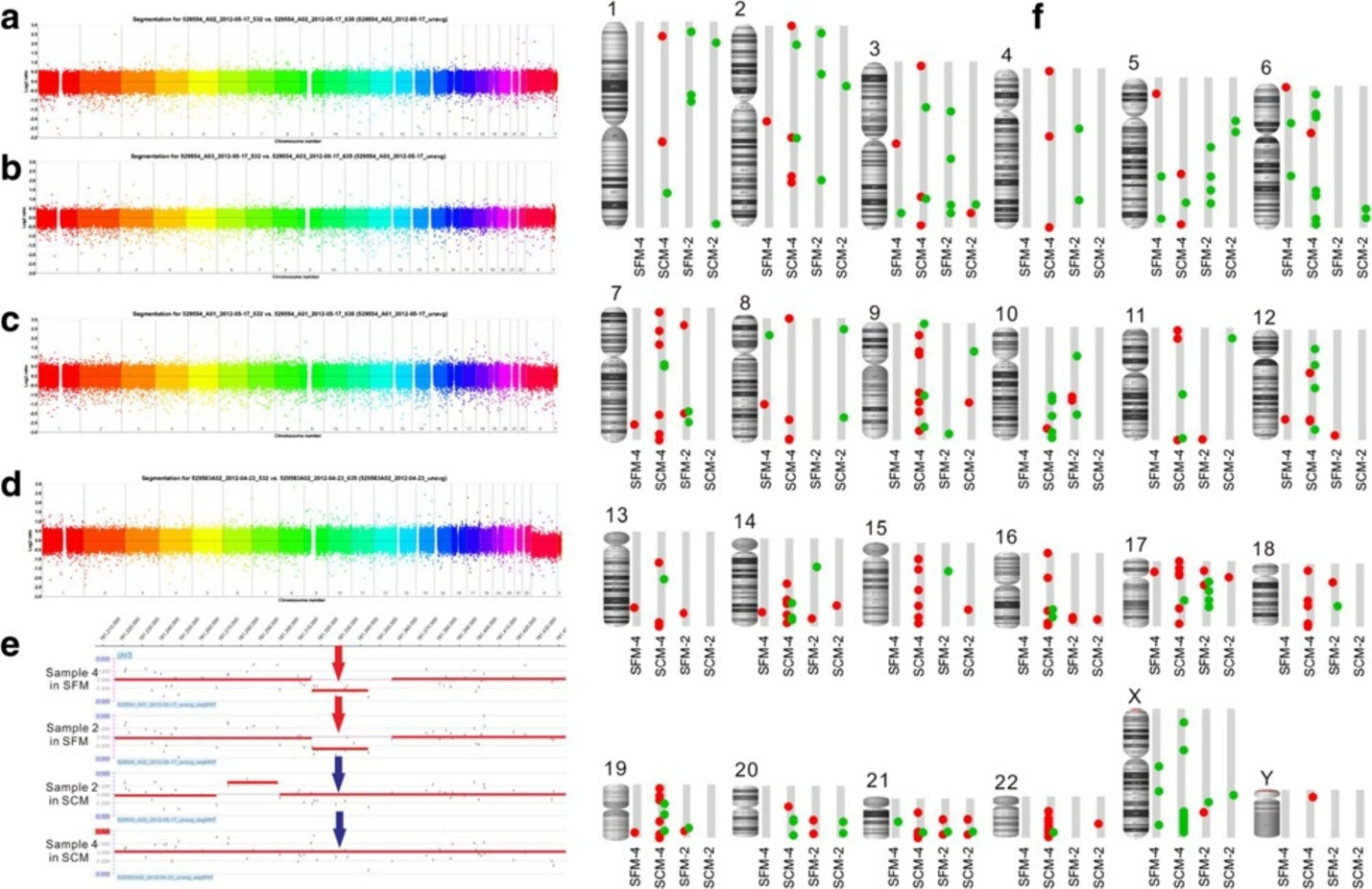
**Array-based comparative genomic hybridization analysis of human umbilical cord mesenchymal stem cells expanded in serum-free medium and serum-containing medium.** Huge unbalanced genome alteration was not obvious in the single-panel rainbow plot, in which each chromosome was differentiated by color. **(a)** Sample 2 in serum-free medium (SFM). **(b)** Sample 2 in serum-containing medium (SCM). **(c)** Sample 4 in SFM. **(d)** Sample 4 in SCM. **(e)** A copy number variation (CNV) segment (chr3:181315609 to 181344028), which was observed in both of the SFM-expanded human umbilical cord mesenchymal stem cells (hUC-MSCs; red arrow), was not observed in either of the SCM-expanded samples (blue arrow). **(f)** CNV identified by array-based comparative genomic hybridization in culture. Amplifications and deletions were mapped onto the human genome for four hUC-MSC clones. Each individual CNV is marked: red circle, amplification; green circle, deletion.

Two pairs of hUC-MSCs (sample 2 and sample 4) at the 10th passage, propagated in SFM and SCM, were assessed by aCGH. Both of the samples showed CNV during *in vitro* culture, no matter whether in SFM or SCM. There was no huge unbalanced genome alteration in SFM-expanded or SCM-expanded hUC-MSCs (Figure 3a,b,c,d). As shown in Table 1 and marked in Figure 3f, a total of 238 CNV segments were observed in this study: 107 segments were gained and the other 131 segments were lost. The length of affected regions varies from 4 kb to 25 Mb, with an average of 154 kb. For sample 2, more CNV was found in SFM than in SCM. However, sample 4 cultured in SCM gained much more CNV than that cultured in SFM. There is therefore no significant difference between SCM-cultured and SFM-cultured MSCs on genomic stability in this study.

**Table 1:** **Summary of CNV observed in SFM-expanded and SCM-expanded hUC-MSCs**

Sample	Deletion		Amplification	
	Number of CNV segments	Sum of CNV length (base pairs)	Number of CNV segments	Sum of CNV length (base pairs)
Sample 4 in SFM	10	319568	11	241958
Sample 4 in SCM	50	27084926	92	7188316
Sample 2 in SFM	31	937255	19	444733
Sample 2 in SCM	16	342620	9	200333

CNV, copy number variation; hUC-MSCs, human umbilical cord mesenchymal stem cells; SCM, serum-containing medium; SFM, serum-free medium.

The loss in chr3:181315609 to 181344028, which was found in both of the SFM-derived hUC-MSCs, did not appear in either of the SCM-expanded hUC-MSCs (Figure 3e). No annotated genes were located in this region, thus the CNV observed only in SFM-expanded hUC-MSCs might have little or no role in altering biological characteristics. Further investigation is needed to study whether the CNV observed only in SFM-expanded hUC-MSCs was implicated, directly or indirectly, in a gene expression regulatory role. Further research was also needed to identify whether the genetic mutation was a consequence of culturing in SFM. In addition, absolute values of the log_2_ ratio of CNV segments observed in this study averaged 0.31, indicating that only a small subpopulation of hUC-MSCs contained these alterations. In other words, hUC-MSCs, at least cultured hUC-MSCs, were a heterogeneous population in the genome.

### Microarray analysis of mRNA in hUC-MSCs expanded in serum-free medium

**Figure 4:**
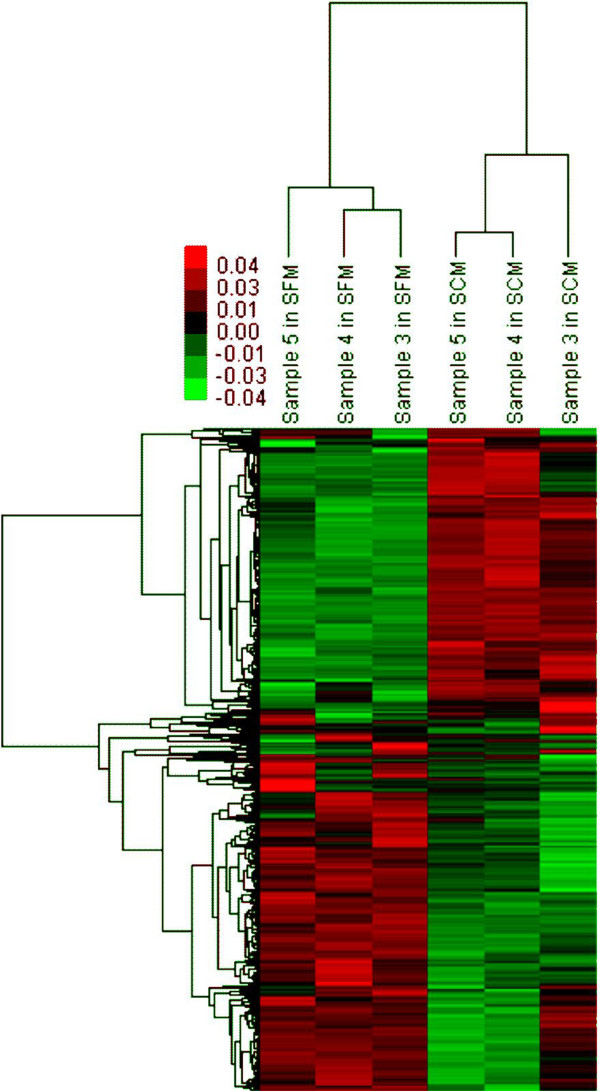
**Clustering of gene expression for serum-free medium-cultured and serum-containing medium-cultured human umbilical cord mesenchymal stem cells.** Two different clusters (expanded in serum-free medium and in serum-containing medium) were quite obvious. Green, downregulation; red, upregulation.

hUC-MSCs from three different donors were analyzed by mRNA chip before and after expansion in SFM. Figure 4 visualizes the differentially expressed genes and correlations between hUC-MSC samples. Six samples were divided into two groups. In the first group, we found all hUC-MSCs cultured in SFM. All hUC-MSCs expanded in SCM fell into the second group. The separation of SFM-expanded hUC-MSCs from SCM-expanded hUC-MSCs was very distinctive.

Gene Ontology analysis of genes with differential expression revealed that cell cycle, mitosis and cell proliferation were changed significantly in SFM-expanded hUC-MSCs (Tables 2 and 3). Similarly, pathway enrichment indicated that many differentially expressed genes in SFM-expanded hUC-MSCs were involved in the cell cycle pathway (Tables 4 and 5). This is consistent with what we observed in *in vitro* culture: hUC-MSCs grow more slowly in SFM compared with growth in SCM.

**Table 2:** **Gene Ontology** (**GO) term enriched by upregulated genes after expansion in serum-free medium (top 10)**

GO term	Count
GO:0006355 regulation of transcription, DNA dependent	57
GO:0006350 transcription	45
GO:0007165 signal transduction	43
GO:0006955 immune response	42
GO:0044419 interspecies interaction between organisms	36
GO:0019882 antigen processing and presentation	30
GO:0002474 antigen processing and presentation of peptide antigen via MHC class I	30
GO:0007275 development	23
GO:0007155 cell adhesion	22
GO:0006468 protein amino acid phosphorylation	21

**Table 3:** **Gene Ontology** (**GO) term enriched by downregulated genes after expansion in serum-free medium (top 10)**

GO term	Count
GO:0007049 cell cycle	92
GO:0006355 regulation of transcription, DNA dependent	87
GO:0006350 transcription	86
GO:0007165 signal transduction	77
GO:0051301 cell division	61
GO:0007067 mitosis	55
GO:0007275 development	52
GO:0006468 protein amino acid phosphorylation	43
GO:0007155 cell adhesion	41
GO:0006260 DNA replication	38

**Table 4:** **Pathways for upregulated genes after expansion in serum-free medium (top 10)**

Pathway	Gene symbol	Count
Regulation of actin cytoskeleton	*ARHGEF6; ARHGEF7; FN1; ITGA10; ITGB8; PDGFD; PIK3R1; PIK3R3; SCIN*	9
Mitogen-activated protein kinase signaling pathway	*DDIT3; ELK4; IL1R1; MAP2K5; MAP2K6; MAP3K5; MAP3K8; MKNK1; PLA2G12A*	9
Systemic lupus erythematosus	*C1R; C1S; HIST1H2AC; HIST1H2BC; HIST1H2BD; HIST1H2BK; HIST1H4H*	7
Jak-STAT signaling pathway	*IL20RB; JAK3; LIFR; PIK3R1; PIK3R3; SPRY1; STAT2*	7
Axon guidance	*CXCL12; EPHA4; EPHB6; SRGAP1; SRGAP3; SRGAP2*	6
Focal adhesion	*FN1; ITGA10; ITGB8; PDGFD; PIK3R1; PIK3R3*	6
Acute myeloid leukemia	*PIK3R1; PIK3R3; PML; RUNX1T1; ZBTB16*	5
Phosphatidylinositol signaling system	*IMPA2; PIK3C2A; PIK3C3; PIK3R1; PIK3R3*	5
Apoptosis	*CFLAR; IL1R1; IRAK4; PIK3R1; PIK3R3*	5
Toll-like receptor signaling pathway	*IRAK4; MAP2K6; MAP3K8; PIK3R1; PIK3R3*	5

**Table 5:** **Pathways for downregulated genes after expansion in serum-free medium (top 10)**

Pathway	Gene symbol	Count
Cell cycle	*BUB1; BUB1B; CCNA2; CCNB1; CCNB2; CCNE2; CDC20; CDC23; CDC25A; CDC25C; CDC45L; CDC6; CDC2; CDK2; CDKN2C; CHEK1; ESPL1; MAD2L1; MCM2; MCM7; MCM3; MCM4; MCM5; MCM7; ORC1L; PLK1; PTTG1; SKP2; SMAD3; TFDP1; TGFB1; TGFB2; WEE1; YWHAH; YWHAZ*	34
Cytokine–cytokine receptor interaction	*CCL2; CCL26; CXCL1; CXCL3; CXCL5; CXCL6; TNFSF13B; EGF; EGFR; FLT1; IL11; IL1A; IL1B; IL6; IL7R; IL8; INHBA; LIF; PDGFC; PF4V1; CCL14; TGFB1; TGFB2; TNFRSF11B; VEGFA*	25
Focal adhesion	*ACTB; COL4A6; COL6A1; EGF; EGFR; FLNB; FLT1; GRLF1; ITGA4; ITGA5; ITGA6; ITGB3; MAPK8; MYLK; PAK2; PDGFC; PDPK1; PRKCA; PTK2; TLN1; TNC; VASP; VEGFA*	23
Regulation of actin cytoskeleton	*ACTB; BDKRB1; DIAPH3; EGF; EGFR; FGF1; FGF2; FGF13; GRLF1; IQGAP3; ITGA4; ITGA5; ITGA6; ITGB3; MYH9; MYLK; NRAS; PAK2; PDGFC; PIP5K1A; PTK2*	21
Mitogen-activated protein kinase signaling pathway	*DUSP4; EGF; EGFR; FGF1; FGF2; FGF13; FLNB; IL1A; IL1B; MAPK8; MAPKAPK2; NRAS; PAK2; PRKACB; PRKCA; RPS6KA3; MAP3K7IP2; TGFB1; TGFB2*	19
DNA polymerase	*FEN1; MCM2; MCM7; MCM3; MCM4; MCM5; MCM7; POLA1; POLA2; POLE2; PRIM1; PRIM2; RFC2; RFC3; RFC4; RFC5; RNASEH2A*	16
Cell adhesion molecules	*CD276; CDH2; CLDN1; ITGA4; ITGA6; JAM3; NEGR1; NEO1; NRXN3; PDCD1LG2; PTPRF; PVR; PVRL2; SDC1; VCAN*	15
Gap junction	*CDC2; EGF; EGFR; GNAS; NRAS; PDGFC; PLCB1; PRKACB; PRKCA; TUBA4A; TUBB2A; TUBB; TUBB2C; TUBB6*	14
Pyrimidine metabolism	*CTPS; POLA1; POLA2; POLE2; POLR2D; POLR3G; PRIM1; PRIM2; RRM1; RRM2; TK1; TYMS; UCK2*	13
Purine metabolism	*GART; PDE7B; PDE8A; PFAS; POLA1; POLA2; POLE2; POLR2D; POLR3G; PRIM1; PRIM2; RRM1; RRM2*	13

**Figure 5:**
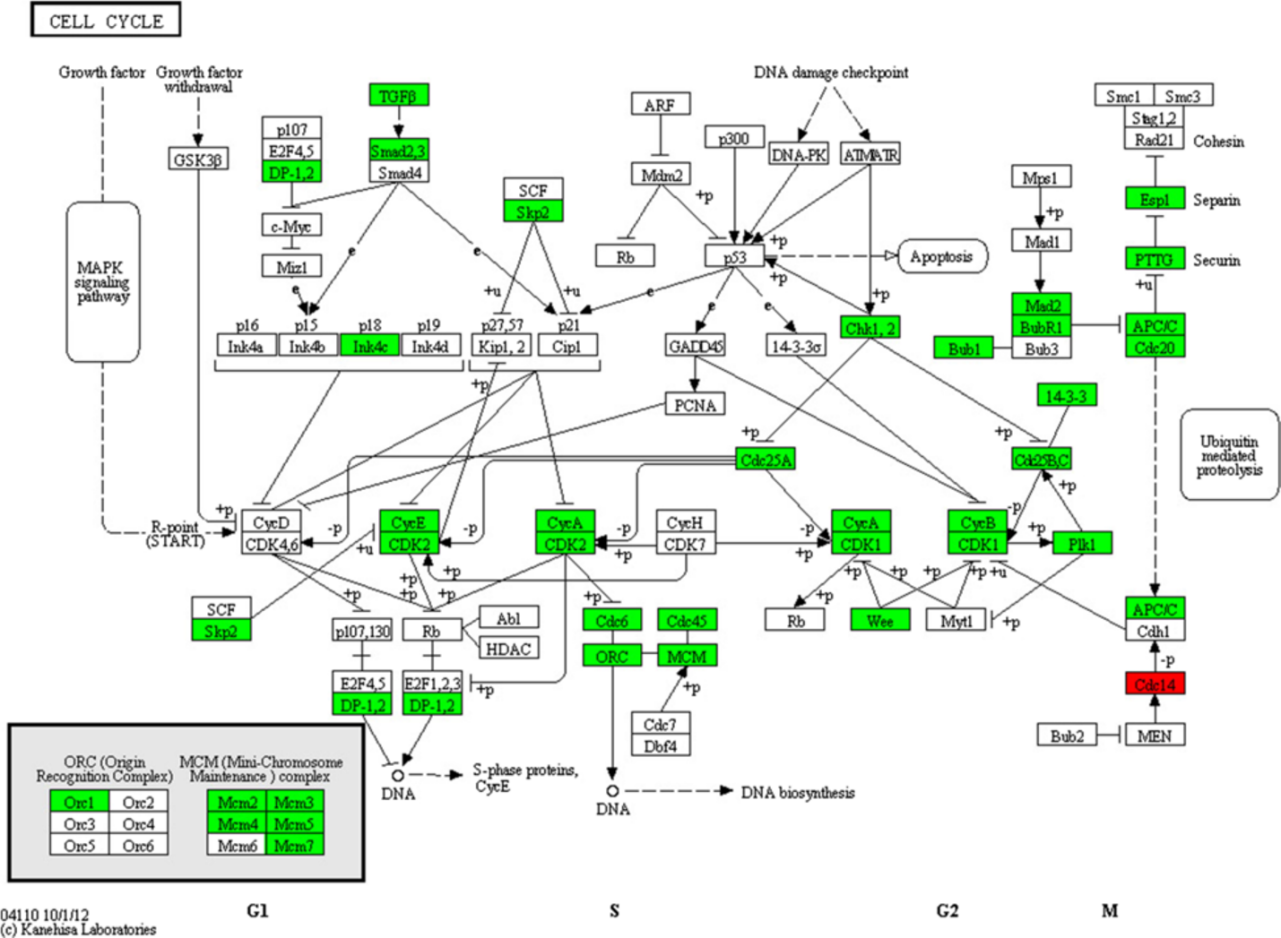
**Upregulated and downregulated genes in the cell cycle pathway after expansion in serum-free medium.** *BUB1*, *BUB1B*, *CCNA2*, *CCNB1*, *CCNB2*, *CCNE2*, *Cdc20*, *Cdc23*, *Cdc25A*, *Cdc25C*, *Cdc45L*, *Cdc6*, *Cdc2*, *CDK2*, *CDKN2C*, *CHEK1*, *ESPL1*, *MAD2L1*, *MCM2*, *MCM7*, *MCM3*, *MCM4*, *MCM5*, *MCM7*, *ORC1L*, *PLK1*, *PTTG1*, *SKP2*, *SMAD3*, *TFDP1*, *TGFB1*, *TGFB2*, *WEE1*, *YWHAH* and *YWHAZ* were downregulated after culture in serum-free medium (SFM). Only *Cdc14* was upregulated during expansion in SFM. Red, genes that were upregulated; green, genes that were downregulated. MAPK, mitogen-activated protein kinase; MCM, Mini-Chromosome Maintenance.

We found that 34 genes in the cell cycle pathway were downregulated (Figure 5) in SFM culture. Many *cyclin* and *cyclin-dependent kinase* (*CDK*) genes were in the list of downregulated genes. Considering that *cyclin* and *CDK* play the role of key regulator in the cell cycle [35], the downregulation of *cyclin* and *CDK* might explain why hUC-MSCs proliferated more slowly in SFM. Besides, we also found that almost all genes in Mini-Chromosome Maintenance (MCM) complex were downregulated in SFM. MCM, as a key component of the prereplication complex, is very important for the initiation and elongation of DNA replication [36, 37]. Downregulation of *MCM* might suppress the synthesis of DNA and then slow the proliferation of hUC-MSCs in SFM. Furthermore, genes encoding growth factor and their receptor, such as *EGF* and *EGFR*, *VEGF* and *VEGFR*, were also downregulated in hUC-MSCs cultured in SFM. Accordingly, a decrease in growth factor and growth factor response might also suppress the growth rate of hUC-MSCs in SFM [38].

Our data also revealed that some genes (*RFC3*, *RFC4*, *RFC5*, *EXO1*, *POLE2*) involved in the mismatch repair and nucleotide excision repair pathway were downregulated in SFM-expanded hUC-MSCs. There are two possible reasons that could explain this phenomenon: instability of genome and reduced growth rate. The aCGH data did not support a striking increase of CNV in SFM-expanded hUC-MSCs, so we cannot blame genome instability for the downregulation of DNA repair-related genes. Reduced growth might thus be a more reasonable explanation. A reduced growth rate led to fewer DNA replications and less DNA errors. So the requirements for mismatch repair and nucleotide excision repair were reduced in SFM-expanded hUC-MSCs. Interestingly, we observed at least five histone-related genes upregulated in SFM-expanded hUC-MSCs: *HIST1H2AC*, *HIST1H2BC*, *HIST1H2BD*, *HIST1H2BK*, *HIST1H4H*. However, we have no idea why histone increased their expression level in SFM-expanded hUC-MSCs, and thus this needs to be further studied.

### Real-time PCR validation

**Figure 6:**
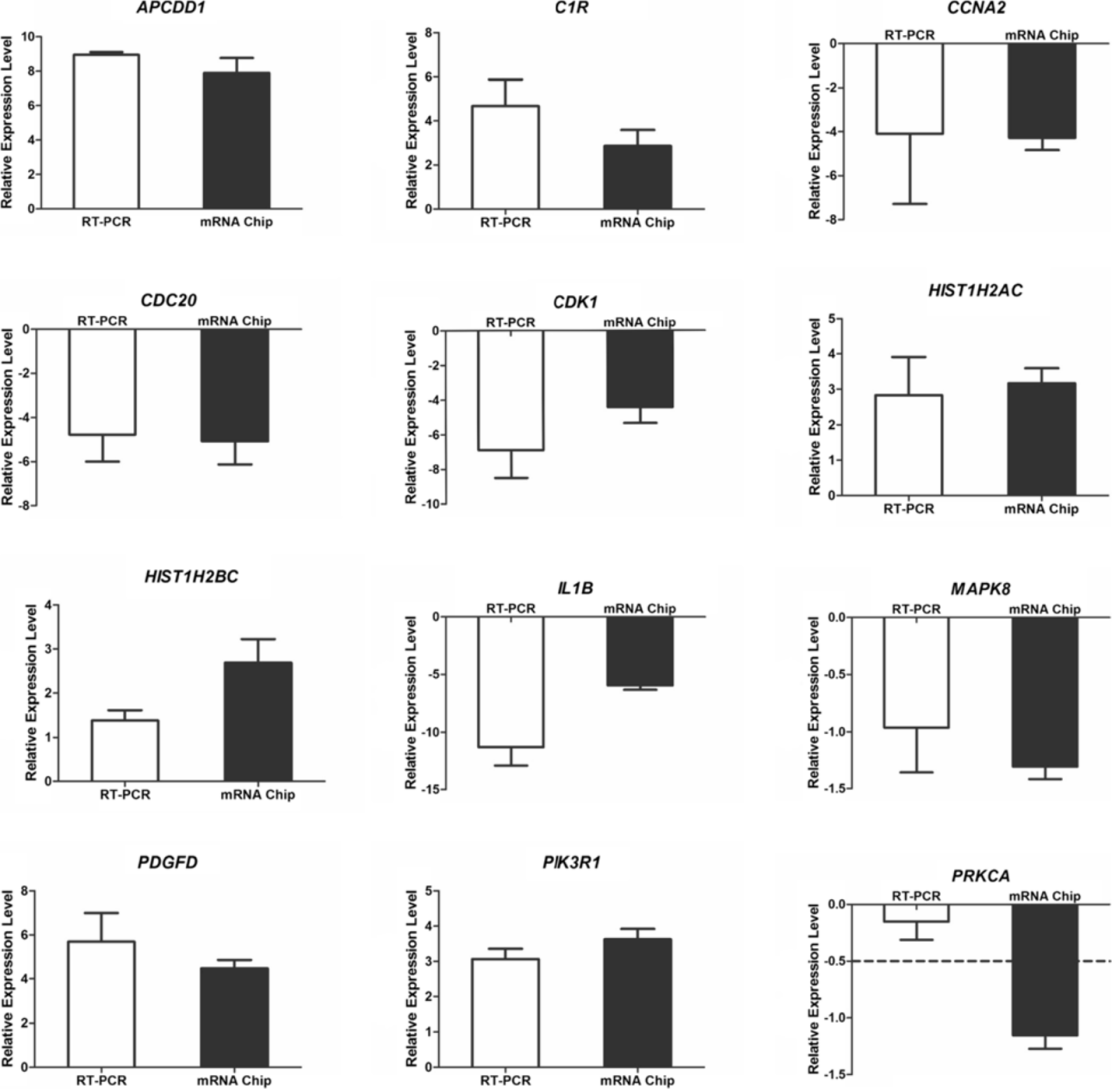
**Validation of the mRNA chip by real-time PCR.** Relative expression levels were calculated by log_2_(fold change): log_2_(fold change) > 1, increasing at least twice; log_2_(fold change) < −1, decreasing at least twice. Among the 12 genes we validated, only *PRKCA* showed an inconsistent expression profile. When analyzed by mRNA chip, the expression of *PRKCA* was downregulated more than twice. However, in the real-time PCR (RT-PCR) analysis, the expression of *PRKCA* did not show a significant difference during the process of long-term culture in serum-free medium.

To confirm and strengthen the findings derived from the mRNA chip, we selected 12 genes and measured their expression level by real-time PCR. As shown in Figure 6, almost all genes analyzed by real-time PCR showed a similar expression profile in the mRNA chip. Real-time PCR data also indicated that genes involved in the cell cycle pathway (*CCNA2*, *CDC20*, *CDK1*) were downregulated, while *HIST1H2AC* and *HIST1H2BC* were upregulated in SFM-expanded hUC-MSCs.

## Discussion

Attaching and proliferating on plastic is a well-described property of MSCs. Fetal bovine serum provides abundant attachment and growth factors that are essential for MSC maintenance and growth. Some SFMs for MSCs are not free of animal-derived components. They need the flask surface to be coated with substrates isolated from animal or human. These substrates are ill-defined and vary significantly between different batches. When compared with animal-derived materials, xeno-free coating substrates that contain components derived from human might be contaminated by communicable pathogens and thus might be more dangerous. While a previous study reported that MSCs may proliferate in three-dimensional cultures without serum and coating substance [39], the three-dimensional system is expensive and not suitable for large-scale expansion to clinical application. To improve cell attachment, a plasma surface treatment is used in *ex vivo* expansion of MSCs. Only in one out of three commercial SFMs provided by different companies in this study can hUC-MSCs attach and propagate well on the plasma-treated surface. Being similar to the bone marrow-derived MSCs cultured in autologous serum [40], hUC-MSCs cultured in SFM require more prolonged exposure to trypsin to dissociate from the plastic surface than hUC-MSCs cultured in SCM. This could be explained, based on the data of transcriptome analysis, by upregulation of many molecules related to cell adhesion (*GPNMB*, *CXCL12*, *COL21A1*, *ITGBL1*). However, we found that hUC-MSCs cultured in SFM could be detached much easier in collagenase compared with trypsin.

Here, we build a system with a xeno-free, serum-free, chemically defined medium and a chemically treated culture surface to expand hUC-MSCs. Neither animal-derived or human-derived protein, nor ill-defined component is included in our culture system. This is a stable, safe, controllable system and thus is suitable for preparing hUC-MSCs for clinical application. The growth rate of hUC-MSCs in this system was lower than in SCM, but all hUC-MSCs in this study could propagate for at least seven passages, which means the amount of hUC-MSCs could be amplified more than 2,000 times (about 11 population doublings) before reaching the senescent stage. If the cell number of the primary culture was 1 × 10^7^[22], we could obtain more than 2 × 10^10^ hUC-MSCs at the seventh passage in SFM, which met the quantity requirement of MSCs for clinical applications.

hUC-MSCs derived from different donors showed a different lifespan in *in vitro* culture. Sometimes, the difference of lifespan between samples was very striking. In the present study, sample 2 maintained proliferative capacities even after 20 passages in SFM. However, sample 4 reached senescence at the 10th passage in SFM. So, we cannot analyze or evaluate SFM for MSCs only in one sample. For fair reproducibility, SFM should be evaluated in MSCs derived from different donors and different tissues.

Considering the close link between genome instability and tumor genesis, whether SFM-expanded MSCs maintain genome stability is also an important safety issue for clinical application. We utilized high-throughput aCGH to evaluate the effect of long-term culture in SFM and in SCM on genomic stability of hUC-MSCs in *in vitro* culture. We found that hUC-MSCs gain copy number changes in culture, no matter whether in SFM or in SCM. This was consistent with the finding in adipose tissue-derived MSCs [15], but differed from the data derived from bone marrow-derived MSCs [33], which might be caused by resolution of different methods. However, our results did not support that hUC-MSCs possess better or worse genome stability in SFM or in SCM.

Tumorigenicity is a most important safety issue when cellular therapies are applied in clinical trials. Many conventional and new technologies provide abundant methods to analyze the genome stability and tumorigenicity. However, Prockop and Keating suggest that ‘the most reliable and simplest test is: do the cells senesce in culture? If they do, they are unlikely to produce tumors or malignancies in patients’ [41]. Previous research has demonstrated that MSCs are unlikely to undergo malignance transformation after long-term culture in SCM [33]. Yet this research did not answer well whether hUC-MSCs were likely to transform or immortalize in SFM. In this study, all hUC-MSC samples derived from five different donors showed growth arrest and senescence in SFM. Thus, we could conclude that hUC-MSCs derived from our culture system, which consisted of SFM and a chemically treated surface, did not undergo malignant transformation.

SFM-expanded hUC-MSCs showed a clear different gene expression profile compared with hUC-MSCs cultured in SCM based on our microarray data. Cell cycle, mitosis and cell proliferation were downregulated in SFM-expanded hUC-MSCs. We observed a low level of *hTERT* in SCM-expanded hUC-MSCs, which cannot be found in SFM-expanded hUC-MSCs. Nevertheless, based on the analysis of cell surface marker and multipotency, SFM-expanded hUC-MSCs met the minimal criteria for human MSCs [29]. The co-culture experiment also revealed that SFM-expanded hUC-MSCs maintained their immunosuppressive ability, which is consistent with previous studies [19, 20]. The change of biological characteristics that was found in SFM-expanded hUC-MSCs might not influence their future clinical applications.

## Conclusions

We studied biological characteristics and therapeutic potential of hUC-MSCs that were expanded in a serum-free and chemically defined culture. Our results indicated that many growth rate-related biological characteristics of hUC-MSCs were altered in SFM. However, SFM-expanded hUC-MSCs met the basic criteria for human MSCs and also maintained immunosuppressive ability. Besides, SFM expansion did not change the genetic stability of hUC-MSCs. SFM-expanded hUC-MSCs are therefore suitable for clinical application.

## Electronic supplementary material

Additional file 1: Figure S1: Showing quantification of *hTERT* and *RPLP0* mRNA in SFM-expanded hUC-MSCs and HeLa (positive control) by TaqMan based real-time PCR. No expression of *hTERT* was detected in SFM-expanded hUC-MSCs derived from five different donors. HeLa were *hTERT*-positive. (JPEG 352 KB)

## Abbreviations

aCGH: array-based comparative genomic hybridization
CDK: cyclin-dependent kinase
CNV: copy number variation
hPBMC: human peripheral blood mononuclear cell
hUC-MSC: human umbilical cord mesenchymal stem cell
IFNγ: interferon gamma
MSC: mesenchymal stem cell
SCM: serum-containing medium
SFM: serum-free medium.
