# Supplementary figures and images for: Human mesenchymal stem cells possess different biological characteristics but do not change their therapeutic potential when cultured in serum free medium
Source: Stem Cell Res Ther. 2014 Dec 4;5(6):132. doi: 10.1186/scrt522 (PMC4445567; doi:10.1186/scrt522)

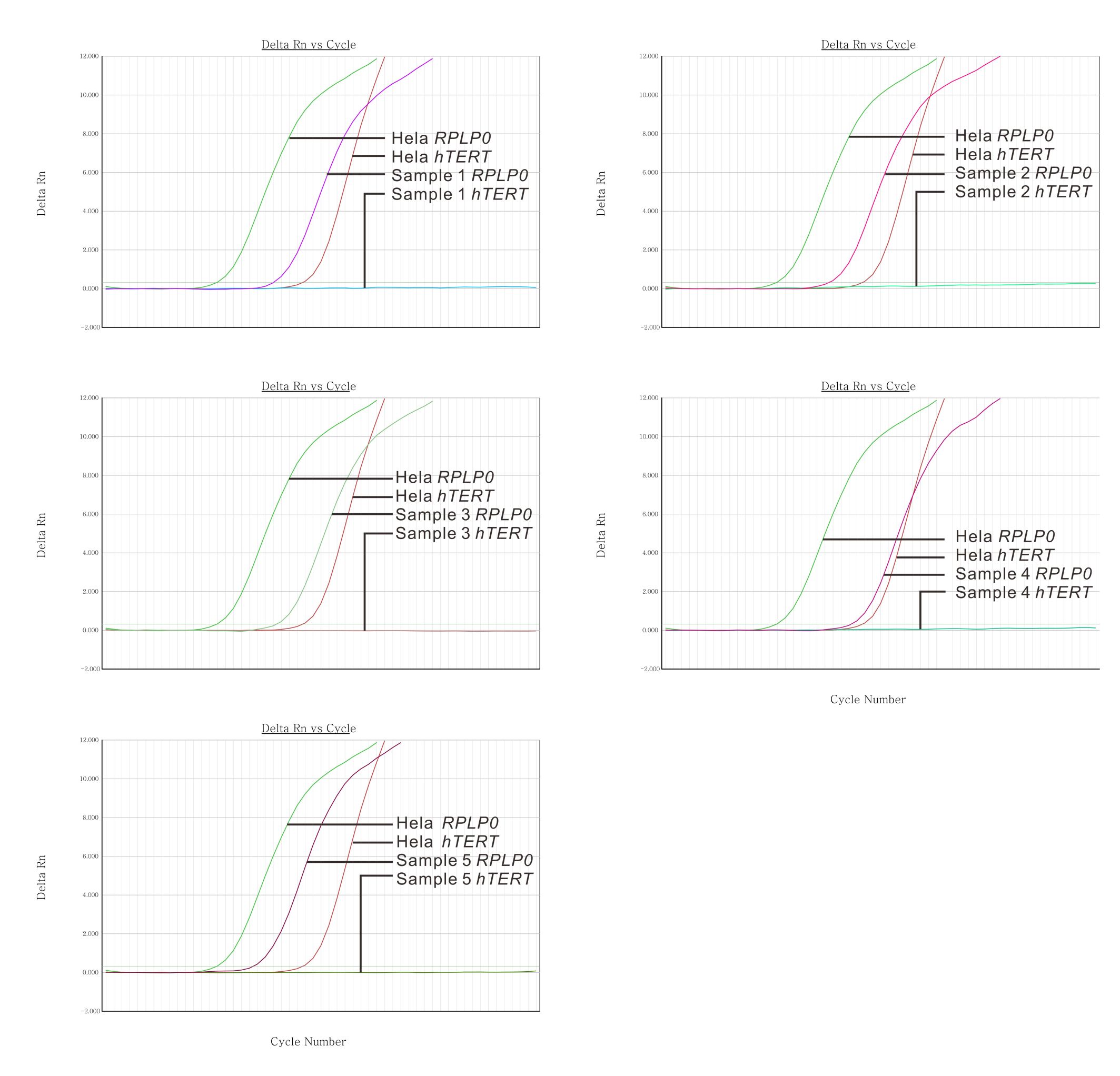

Supplement: Supplementary file 1 — Additional file 1: Figure S1: Showing quantification of hTERT and RPLP0 mRNA in SFM-expanded hUC-MSCs and HeLa (positive control) by TaqMan based real-time PCR. No expression of hTERT was detected in SFM-expanded hUC-MSCs derived from five different donors. HeLa were hTERT-positive. (JPEG 352 KB) [file 13287_2014_442_MOESM1_ESM.jpeg]
